# Ethnic differences in the incidence of clinically diagnosed influenza: an England population-based cohort study 2008-2018

**DOI:** 10.12688/wellcomeopenres.16620.2

**Published:** 2021-05-05

**Authors:** Jennifer Davidson, Amitava Banerjee, Rohini Mathur, Mary Ramsay, Liam Smeeth, Jemma Walker, Helen McDonald, Charlotte Warren-Gash

**Affiliations:** 1 Department of Non-communicable Disease Epidemiology, London School of Hygiene and Tropical Medicine, London, UK; 2 Institute of Health Informatics, University College London, London, UK; 3 Immunisation and Countermeasures, National Infection Service, Public Health England, London, UK; 4 National Institute for Health Research Health Protection Research Unit in Immunisation, London School of Hygiene and Tropical Medicine in partnership with Public Health England, London, UK; 5 Department of Infectious Disease Epidemiology, London School of Hygiene and Tropical Medicine, London, UK; 6 Statistics, Modelling and Economics Department, Public Health England, London, UK

**Keywords:** ethnicity, inequalities, influenza, respiratory infection

## Abstract

**Background:** People of non-White ethnicity have a higher risk of severe outcomes following influenza infection. It is unclear whether this is driven by an increased risk of infection or complications. We therefore aimed to investigate the incidence of clinically diagnosed influenza/influenza-like illness (ILI) by ethnicity in England from 2008-2018.

**Methods:** We used linked primary and secondary healthcare data (from the Clinical Practice Research Datalink [CPRD] GOLD and Aurum databases and Hospital Episodes Statistics Admitted Patient Care [HES APC]). We included patients with recorded ethnicity who were aged 40-64 years and did not have a chronic health condition that would render them eligible for influenza vaccination. ILI infection was identified from diagnostic codes in CPRD and HES APC. We calculated crude annual infection incidence rates by ethnic group. Multivariable Poisson regression models with random effects were used to estimate any ethnic disparities in infection risk. Our main analysis adjusted for age, sex, and influenza year.

**Results:** A total of 3,735,308 adults aged 40-64 years were included in the study; 87.6% White, 5.2% South Asian, 4.2% Black, 1.9% Other, and 1.1% Mixed. We identified 102,316 ILI episodes recorded among 94,623 patients. The rate of ILI was highest in the South Asian (9.6 per 1,000 person-years), Black (8.4 per 1,000 person-years) and Mixed (6.9 per 1,000 person-years) ethnic groups. The ILI rate in the White ethnic group was 5.7 per 1,000 person-years. After adjustment for age sex and influenza year, higher incidence rate ratios (IRR) for ILI were seen for South Asian (1.70, 95% CI 1.66-1.75), Black (1.48, 1.44-1.53) and Mixed (1.22, 1.15-1.30) groups compared to White ethnicity.

**Conclusions:** Our results suggest that influenza infection risk differs between White and non-White groups who are not eligible for routine influenza vaccination.

## Introduction

People from ethnic minority backgrounds are represented disproportionately among patients with severe coronavirus disease 2019 (COVID-19). Early in the pandemic there were reports of excess COVID-related critical care admissions and deaths among people from Black and South Asian ethnic groups ^1, 2^. Recent research has found people of Black and South Asian ethnicity have increased risk of severe acute respiratory syndrome coronavirus 2 (SARS-CoV-2) infection, COVID-19-related hospitalization and death, independent of deprivation, occupation, household size, and underlying health conditions ^3, 4^.

The COVID-19 pandemic has reinforced the importance of seasonal influenza vaccination. By preventing influenza-related hospitalization, vaccination can minimize the risk of hospital-acquired COVID-19 (co-) infection for these individuals and reduce health service pressures, particularly the need for isolation of patients with respiratory symptoms awaiting COVID-19 test results.

In the United Kingdom (UK), the influenza vaccine is routinely recommended for adults aged ≥65 years, or people <65 years with underlying health conditions. These recommendations formed the basis of the original guidance to identify patients at moderate- and high-risk of COVID-19. Influenza vaccine recommendations were expanded for the 2020/21 season to include all adults ≥50 years ^5^. However, vaccine uptake among clinical risk groups is low, particularly for Black and Mixed Black ethnic groups ^6^. This is consistent with previous findings that Black ethnicity is associated with lower influenza vaccine uptake among children and pregnant women ^7– 9^. In addition, people of non-White ethnicity have higher risk of severe outcomes following influenza infection ^10, 11^. It is unclear whether this is driven by the risk of infection or complications, with most research focused on distal outcomes rather than initial infection risk.

Here we use clinical diagnoses data to investigate the incidence of influenza and influenza-like illness (ILI) by ethnicity from 2008–2018 among people not eligible for routine influenza vaccination, to consider disparities in infection risk.

## Methods

### Study design and data sources

We conducted a retrospective cohort study using anonymized primary care data from the UK Clinical Practice Research Datalink (CPRD) GOLD and Aurum databases ^12, 13^ with linked secondary care data from the Hospital Episodes Statistics Admitted Patient Care (HES APC) database and death data from the Office for National Statistics. CPRD GOLD and Aurum collect records from >35 million patients registered at with National Health Service (NHS) general practitioners. The data include diagnoses, prescriptions, immunizations and demographics. HES APC data are collated from inpatient care at all NHS hospitals in England. The NHS is a universal health system publicly funded through general taxation which is accessible to all UK residents, although there is an annual surcharge for people who move to the UK. The corresponding author had full access to all CPRD GOLD and Aurum data used in the study, with relevant linked patient HES APC and ONS death data obtained from CPRD.

### Study population

We included all adults aged 40–64 years registered at a CPRD contributing practice in England between 01/09/2008 and 31/08/2018 who were present in the GOLD and Aurum datasets. We then excluded among this study population any patients with a health condition indicative of influenza vaccination eligibility ( Table 1), and those who had ever received pneumococcal vaccination, or influenza vaccination in the 12 months before baseline (all codes listed here, DOI: https://doi.org/10.17037/DATA.00002102). Among the final study population, we started study follow-up to identify diagnoses of ARI (outcome of interest) at the latest of 12 months after current registration, up-to-research-standard (GOLD only), 40 ^th^ birthday, or 01/09/2008. Follow-up ended at the earliest of; a new diagnosis of a condition conferring eligibility for vaccination, pneumococcal or influenza vaccination, death, transfer out, the practice’s last data collection, 65th birthday, or 31/08/2018.

**Table 1.** Definitions used for developing exclusion conditions using Clinical Practice Research Datalink (CPRD) code lists.

Health condition	Study definition
Cardiovascular disease (CVD)	Any previous clinical diagnosis, major intervention for, or clinical review specific to CVD including heart disease (congenital or otherwise), heart failure, stroke or transient ischaemic attack.
Chronic liver disease	Any previous clinical diagnosis of, or clinical review specific to, chronic liver disease including cirrhosis, oesophageal varices, biliary atresia and chronic hepatitis.
Chronic kidney disease (CKD)	Any previous clinical diagnosis of, or clinical review specific to, CKD stages 3–5, history of dialysis or renal transplant in Gold or Aurum. Or with estimated glomerular filtration rate (eGFR) to classify CKD stage 3–5 in Gold.
Chronic respiratory disease	Any previous clinical diagnosis of, or clinical review specific to, chronic respiratory disease, including chronic obstructive pulmonary disease, emphysema, bronchitis, cystic fibrosis, or fibrosing interstitial lung diseases.
Asthma	Any previous clinical diagnosis of, or clinical review specific to, asthma with at least two prescriptions of inhaled steroids in the year before baseline. Or any previous hospitalisation for asthma.
Chronic neurological disease	Any previous clinical diagnosis of, or clinical review specific to, a neurological disease such as Parkinson’s disease, motor neurone disease, multiple sclerosis (MS), cerebral palsy, dementia or a learning/ intellectual disability.
Diabetes mellitus	Any previous diagnosis of, or clinical review specific to, diabetes mellitus, or with a prescription for medication used to treat diabetes.
Asplenia/sickle cell disease	Any previous clinical diagnosis of, or clinical review specific to, asplenia or dysfunction of the spleen (including sickle cell disease but not sickle cell trait).
Severe obesity	Latest body mass index before baseline was ≥40 kg/m ^2^.
Immunosuppression	Any previous clinical diagnosis of, or clinical review specific to, HIV, solid organ transplant or other permanent immunosuppression (such as genetic conditions compromising immune function).
	In the two years before baseline: clinical diagnosis of, or clinical review specific to, aplastic anaemia or haematological malignancy, or receiving a bone marrow or stem cell transplant.
	In the year before baseline: previous clinical diagnosis of, or clinical review specific to, other/unspecified immune deficiency or receiving chemotherapy or radiotherapy.
	In the year before baseline: prescription of biological therapy or at least 2 prescriptions for oral steroids or other immunosuppressants including DMARDS, Methotrexate, Azathioprine, or corticosteroid injections.

### Variables

Our exposure of self-reported ethnicity was captured in CPRD and supplemented with HES APC if missing in CPRD. We grouped ethnicity into the five and 16 census categories (the relevant subgroups from the 16 categorization are shown after the corresponding five category group in brackets in the following list) of White (British, Irish, Other White), South Asian (Indian, Pakistani, Bangladeshi, other Asian), Black (African, Caribbean, other Black), Other (Chinese, all other), and Mixed (White and Asian, White and African, White and Caribbean, Other Mixed).

Our outcome of influenza/ILI was identified from diagnostic codes in CPRD and HES APC. In a second analysis, we expanded our outcome definition to acute respiratory infection (ARI), additionally including codes for pneumonia, acute bronchitis, or other acute infections suggestive of lower respiratory tract involvement (all codes listed here, DOI: https://doi.org/10.17037/DATA.00002102). We considered the following confounders in our analysis; age (grouped into 40–44, 45–49, 50–54, 55–59 and 60–64), sex (men or women), year of outcome, region of residence and socioeconomic status. Region of residence was classified using the 10 regionally breakdowns for England available within CPRD. Socioeconomic status was assigned based on Townsend score quintile.

### Statistical analysis

All analyses were done with Stata (version 16). We calculated crude annual infection incidence rates by ethnic group with age- and sex-stratification. Multivariable Poisson regression models with random effects, to account for multiple infections in the same patient, were used to estimate any ethnic disparities in infection risk. Our main analysis adjusted for age, sex, and influenza season/year. A second model additionally adjusted for region of residence and socioeconomic status, which may both confound and mediate an association between ethnicity and infection. Influenza circulation may vary regionally with the ethnic profile of the population also varying by region. Socioeconomic disadvantage is a risk factor for many infectious diseases with socioeconomic disadvantage also more prevalent in non-White ethnic groups in England.

An earlier version of this article can be found on medRxiv ( https://doi.org/10.1101/2021.01.15.21249388).

## Results

**Figure 1.**
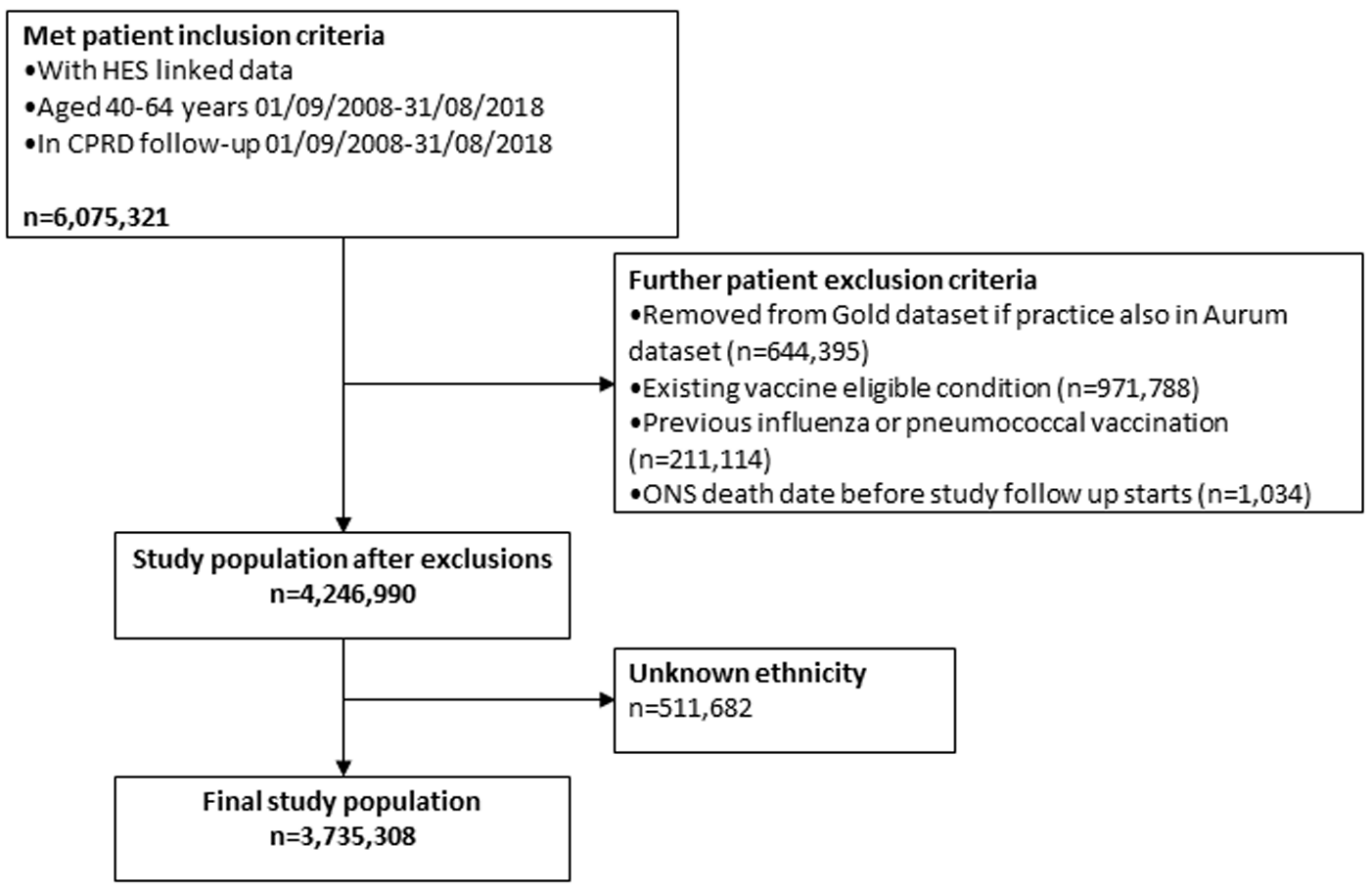
Study population flow chart.

Our cohort included 3,735,308 patients ( Figure 1), of whom 87.6% were White (n=3,271,115), 5.2% South Asian (n=196,262), 4.2% Black (n=157,075), 1.9% Other (n=69,440), and 1.1% Mixed (n=41,416) ( Table 2). We excluded 511,682 (12.0%) patients with no recorded ethnicity; this group had longer follow-up, fewer consultations and were more likely to be male than the included study population ( Table 2). 16-category ethnicity was known for 3,035,689 of the cohort (with HES ethnicity breakdown beyond white and mixed not available), of whom 76.3% were White British, 0.9% Irish, 7.7% Other White, 2.6% Indian, 1.3% Pakistani, 0.4% Bangladeshi, 2.2% Other Asian, 2.8% African, 1.5% Caribbean, 0.9% Other Black, 0.7% Chinese, and 1.5% Other ( Table 3). Non-White populations were younger and resided in more deprived areas than the White population, while a higher proportion of the White population were obese.

**Table 2.** Baseline characteristics by five category ethnic group.

	All (N=3,735,308)	White (N=3,271,115)	South Asian (N=196,262)	Black (N=157,075)	Other (N=69,440)	Mixed (N=41,416)	Unknown (N=511,682)
Median (IQR) length of CPRD follow-up	3.8 (1.5-7.4)	4.0 (1.6-7.8)	2.9 (1.2-6.0)	3.2 (1.2-3.2)	2.7 (1.1-5.6)	3.0 (1.2-6.1)	4.2 (1.8-8.2)
Median (IQR) age in years	46 (40–54)	46 (40–54)	42 (40–49)	44 (40–49)	44 (40–50)	43 (40–49)	45 (40–52)
Age (years)							
40–44	1,685,587 (45.1%)	1,413,858 (43.2%)	120,070 (61.2%)	88,521 (56.4%)	38,703 (55.7%)	24,435 (59.0%)	238,146 (46.5%)
45–49	687,429 (18.4%)	601,812 (18.4%)	31,297 (15.9%)	33,344 (21.2%)	12,959 (18.7%)	8,017 (19.4%)	98,669 (19.3%)
50–54	540,921 (14.5%)	485,662 (14.8%)	21,793 (11.1%)	19,969 (12.7%)	8,685 (12.5%)	4,812 (11.6%)	76,388 (14.9%)
55–59	442,898 (11.9%)	410,176 (12.5%)	14,480 (7.4%)	10,020 (6.4%)	5,594 (8.1%)	2,628 (6.3%)	56,292 (11.0%)
60–64	378,473 (10.1%)	359,607 (11.0%)	8,622 (4.4%)	5,221 (3.3%)	3,499 (5.0%)	1,524 (3.7%)	42,187 (8.2%)
Sex (N=3,735,282)							
Male	1,881,393 (50.4%)	1,643,111 (50.2%)	101,539 (51.7%)	81,875 (52.1%)	34,067 (49.1%)	20,801 (50.2%)	356,673 (69.7%)
Female	1,853,889 (49.6%)	1,627,982 (49.8%)	94,719 (48.3%)	75,200 (47.9%)	35,373 (50.9%)	20,615 (49.8%)	155,003 (30.3%)
Townsend quintile (N=3,731,066)							
1 (least deprived)	880,963 (23.6%)	837,637 (25.6%)	23,103 (11.8%)	5,925 (3.8%)	9,291 (13.4%)	5,007 (12.1%)	132,214 (25.9%)
2	799,291 (21.4%)	756,591 (23.2%)	21,042 (10.7%)	7,311 (4.7%)	9,131 (13.2%)	5,216 (12.6%)	113,630 (22.3%)
3	730,372 (19.6%)	663,347 (20.3%)	34,353 (17.5%)	14,337 (9.1%)	11,648 (16.8%)	6,687 (16.2%)	101,845 (19.9%)
4	658,321 (17.6%)	549,667 (16.8%)	52,631 (26.8%)	32,303 (20.6%)	14,817 (214%)	8,903 (21.5%)	86,739 (17.0%)
5 (most deprived)	662,119 (17.7%)	460,004 (14.1%)	65,030 (33.2%)	97,043 (61.8%)	24,475 (35.3%)	15,567 (37.6%)	76,123 (14.9%)
Region of residence in England							
North East	147,068 (3.9%)	141,750 (4.3%)	2,440 (1.2%)	936 (0.6%)	1,251 (1.8%)	691 (1.7%)	12,166 (2.4%)
North West	565,744 (15.1%)	523,858 (16.0%)	18,809 (9.6%)	9,303 (5.9%)	9,473 (13.7%)	4,301 (10.4%)	65,729 (12.9%)
Yorkshire & Humber	154,636 (4.1%)	147,769 (4.5%)	3,072 (1.6%)	1,429 (0.9%)	1,478 (2.1%)	888 (2.1%)	18,839 (3.7%)
East Midlands	100,776 (2.7%)	92,052 (2.8%)	4,687 (2.4%)	2,013 (1.3%)	1,238 (1.8%)	786 (1.9%)	16,320 (3.2%)
West Midlands	583,179 (15.6%)	517,656 (15.8%)	39,308 (20.1%)	15,130 (9.6%)	6,292 (9.1%)	4,793 (11.6%)	65,633 (12.8%)
East of England	266,808 (7.1%)	244,371 (7.5%)	10,830 (5.5%)	5,390 (3.4%)	3,822 (5.5%)	2,395 (5.8%)	49,571 (9.7%)
South West	519,561 (13.9%)	492,718 (15.1%)	9,083 (4.6%)	8,282 (5.3%)	5,648 (8.1%)	3,830 (9.3%)	76,978 (15.0%)
South Central	452,647 (12.1%)	415,662 (12.7%)	18,209 (9.3%)	7,493 (4.8%)	7,128 (10.3%)	4,155 (10.0%)	73,726 (14.4%)
London	634,621 (17%)	409,709 (12.5%)	77,962 (39.8%)	102,364 (65.2%)	27,818 (40.1%)	16,768 (40.5%)	72,056 (14.1%)
South East	309,661 (8.3%)	285,441 (8.7%)	11,566 (5.9%)	4,611 (2.9%)	5,248 (7.6%)	2,795 (6.8%)	60,469 (11.8%)
Median (IQR) consultations in prior 12 months	3 (1-7)	3 (1-7)	4 (1-8)	4 (1-8)	2 (0-6)	3 (1-7)	0 (0-2)
BMI category * (N=2,850,103)							
Underweight	45,771 (1.5%)	37,972 (1.4%)	3,988 (2.3%)	1,375 (1%)	1,765 (3.2%)	671 (1.9%)	5,510 (1.7%)
Normal weight	1,305,069 (41.4%)	1,145,430 (41.6%)	74,311 (43.2%)	41,008 (30.2%)	29,338 (52.5%)	14,982 (42.9%)	153,692 (47.5%)
Overweight	1,170,870 (37.1%)	1,019,097 (37.0%)	66,627 (38.7%)	54,580 (40.2%)	17,859 (31.9%)	12,707 (36.3%)	116,081 (35.9%)
Obese	633,638 (20.1%)	553,921 (20.1%)	27,192 (15.8%)	38,960 (28.7%)	6,966 (12.5%)	6,599 (18.9%)	48,334 (14.9%)
Smoking status * (N=1,369,548)							
Non-smoker	1,491,210 (40.5%)	1,238,035 (38.4%)	112,955 (58.3%)	83,459 (54%)	38,148 (56.7%)	18,613 (45.7%)	208,281 (48.1%)
Current smoker	965,354 (26.2%)	874,223 (27.1%)	32,779 (16.9%)	33,543 (21.7%)	13,987 (20.8%)	10,822 (26.6%)	117,923 (27.3%)
Ex-smoker	1,227,640 (33.3%)	1,115,714 (34.6%)	47,855 (24.7%)	37,644 (24.3%)	15,109 (22.5%)	11,318 (27.8%)	106,393 (24.6%)
Alcohol use * (N=1,087,268)							
Not a heavy drinker	3,171,941 (94.4%)	2,773,635 (41%)	172,349 (97.1%)	135,867 (96.1%)	55,538 (96.9%)	34,552 (95.1%)	336,752 (96.6%)
Heavy drinker	186,656 (5.6%)	172,369 (5.9%)	5,192 (2.9%)	5,521 (3.9%)	1,777 (3.1%)	1,797 (4.9%)	11,680 (3.4%)

*Closest measure before baseline. BMI; body mass index, CPRD; Clinical Practice Research Datalink, IQR; interquartile range

**Table 3.** Baseline characteristics by 16 category ethnic group.

	British (N=2,315,904)	Irish (N=2,315,904)	Other White (N=232,692)	Caribbean (N=44,722)	African (N=84,355)	Other Black (N=27,998)	Chinese (N=23,419)	Other (N=46,021)
Median (IQR) length of CPRD follow-up	4.2 (1.8-8.1)	3.4 (1.3-7.1)	2.3 (1.0-5.0)	4.1 (1.5-7.8)	2.8 (1.2-5.4)	3.2 (1.2-6.3)	3.1 (1.2-6.2)	2.5 (1.0-5.3)
Median (IQR) age in years	47 (41-54)	47 (41-55)	43 (40-50)	45 (40-51)	43 (40-48)	44 (40-49)	44 (40-51)	43 (40-50)
Age (years)								
40–44	975,830 (42.1%)	11,709 (42.8%)	134,661 (57.9%)	21,314 (47.7%)	51,138 (60.6%)	16,069 (57.4%)	12,569 (53.7%)	26,134 (56.8%)
45–49	425,775 (18.4%)	4,788 (17.5%)	40,182 (17.3%)	10,616 (23.7%)	16,491 (19.5%)	6,237 (22.3%)	4,432 (18.9%)	8,527 (18.5%)
50–54	351,291 (15.2%)	3,993 (14.6%)	27,172 (11.7%)	7,158 (16%)	9,426 (11.2%)	3,385 (12.1%)	3,097 (13.2%)	5,588 (12.1%)
55–59	299,319 (12.9%)	3,608 (13.2%)	18,688 (8%)	3,705 (8.3%)	4,754 (5.6%)	1,561 (5.6%)	2,038 (8.7%)	3,556 (7.7%)
60–64	263,689 (11.4%)	3,241 (11.9%)	11,989 (5.2%)	1,929 (4.3%)	2,546 (3%)	746 (2.7%)	1,283 (5.5%)	2,216 (4.8%)
Sex								
Male	1,164,363 (50.3%)	14,595 (53.4%)	115,171 (49.5%)	21,648 (48.4%)	45,472 (53.9%)	14,755 (52.7%)	10,378 (44.3%)	23,689 (51.5%)
Female	1,151,524 (49.7%)	12,744 (46.6%)	117,519 (50.5%)	23,074 (51.6%)	38,883 (46.1%)	13,243 (47.3%)	13,041 (55.7%)	22,332 (48.5%)
Townsend quintile								
1 (least deprived)	605,879 (26.2%)	4,177 (15.3%)	28,234 (12.1%)	1,699 (3.8%)	2,889 (3.4%)	1,337 (4.8%)	3,851 (16.5%)	5,440 (11.8%)
2	549,739 (23.8%)	4,451 (16.3%)	31,493 (13.5%)	2,031 (4.5%)	3,666 (4.3%)	1,614 (5.8%)	3,517 (15%)	5,614 (12.2%)
3	474,256 (20.5%)	5,074 (18.6%)	40,302 (17.3%)	4,442 (9.9%)	6,963 (8.3%)	2,932 (10.5%)	4,282 (18.3%)	7,366 (16%)
4	380,010 (16.4%)	6,112 (22.4%)	53,835 (23.2%)	9,781 (21.9%)	16,625 (19.7%)	5,897 (21.1%)	5,105 (21.8%)	9,712 (21.1%)
5 (most deprived)	303,831 (13.1%)	7,504 (27.5%)	78,660 (33.8%)	26,717 (59.8%)	54,134 (64.2%)	16,192 (57.9%)	6,640 (28.4%)	17,835 (38.8%)
Region of residence in England								
North East	113,825 (4.9%)	277 (1%)	3,369 (1.4%)	71 (0.2%)	680 (0.8%)	185 (0.7%)	558 (2.4%)	693 (1.5%)
North West	389,948 (16.8%)	3,272 (12%)	16,070 (6.9%)	2,067 (4.6%)	4,758 (5.6%)	2,478 (8.9%)	3,563 (15.2%)	5,910 (12.8%)
Yorkshire & Humber	110,564 (4.8%)	407 (1.5%)	4,709 (2%)	237 (0.5%)	909 (1.1%)	283 (1%)	579 (2.5%)	899 (2%)
East Midlands	63,310 (2.7%)	349 (1.3%)	3,627 (1.6%)	742 (1.7%)	946 (1.1%)	325 (1.2%)	684 (2.9%)	554 (1.2%)
West Midlands	401,011 (17.3%)	3,171 (11.6%)	22,389 (9.6%)	5,978 (13.4%)	6,203 (7.4%)	2,949 (10.5%)	2,634 (11.3%)	3,658 (8%)
East of England	159,626 (6.9%)	2,024 (7.4%)	12,727 (5.5%)	1,468 (3.3%)	2,904 (3.4%)	1,018 (3.6%)	1,424 (6.1%)	2,398 (5.2%)
South West	341,551 (14.7%)	2,078 (7.6%)	22,475 (9.7%)	2,374 (5.3%)	3,789 (4.5%)	2,119 (7.6%)	1,941 (8.3%)	3,707 (8.1%)
South Central	295,742 (12.8%)	2,367 (8.7%)	25,541 (11%)	1,776 (4%)	4,187 (5%)	1,530 (5.5%)	2,542 (10.9%)	4,586 (10%)
London	252,414 (10.9%)	11,564 (42.3%)	106,743 (45.9%)	29,070 (65.1%)	57,177 (67.8%)	16,117 (57.6%)	7,968 (34.1%)	19,850 (43.2%)
South East	187,857 (8.1%)	1,822 (6.7%)	15,003 (6.4%)	896 (2%)	2,736 (3.2%)	979 (3.5%)	1,506 (6.4%)	3,742 (8.1%)
Median (IQR) consultations in prior 12 months	3 (1-7)	3 (1-7)	3 (1-6)	4 (1-8)	3 (1-8)	4 (1-8)	2 (0-5)	3 (0-7)
BMI category *								
Underweight	25,949 (1.3%)	294 (1.3%)	3,256 (1.6%)	450 (1.2%)	632 (0.9%)	293 (1.2%)	977 (5%)	788 (2.2%)
Normal weight	815,240 (40.9%)	10,000 (42.5%)	89,109 (44.8%)	13,004 (33.6%)	20,065 (27.4%)	7,939 (33.3%)	13,220 (68.1%)	16,118 (44.1%)
Overweight	743,349 (37.3%)	8,888 (37.8%)	71,860 (36.2%)	14,780 (38.1%)	30,494 (41.6%)	9,306 (39%)	4,466 (23%)	13,393 (36.7%)
Obese	406,582 (20.4%)	4,338 (18.4%)	34,468 (17.3%)	10,522 (27.1%)	22,131 (30.2%)	6,307 (26.4%)	743 (3.8%)	6,223 (17%)
Smoking status *								
Non-smoker	870,495 (37.9%)	9,075 (33.5%)	90,175 (39.5%)	18,082 (40.9%)	52,028 (62.8%)	13,349 (48.4%)	14,593 (63.8%)	23,555 (53.1%)
Current smoker	605,095 (26.3%)	8,208 (30.3%)	67,639 (29.6%)	14,367 (32.5%)	11,693 (14.1%)	7,483 (27.1%)	3,484 (15.2%)	10,503 (23.7%)
Ex-smoker	823,529 (35.8%)	9,803 (36.2%)	70,349 (30.8%)	11,729 (26.5%)	19,180 (23.1%)	6,735 (24.4%)	4,802 (21%)	10,307 (23.2%)
Alcohol consumption *								
Not a heavy drinker	2,005,523 (93.9%)	22,756 (90.8%)	190,358 (95.1%)	39,216 (95.6%)	73,011 (96.7%)	23,640 (95.1%)	19,310 (97.6%)	36,228 (96.5%)
Heavy drinker	130,642 (6.1%)	2,303 (9.2%)	9,884 (4.9%)	1,797 (4.4%)	2,494 (3.3%)	1,230 (4.9%)	469 (2.4%)	1,308 (3.5%)
	**Indian** **(N=79,409)**	**Pakistani** **(N=39,059)**	**Bangladeshi** **(N=12,095)**	**Other Asian** **(N=65,699)**	**White + Black Caribbean** **(N=7,298)**	**White + Black African** **(N=7,663)**	**White + Asian** **(N=7,060)**	**Other Mixed** **(N=14,956)**
Median (IQR) length of CPRD follow-up	3.2 (1.2-6.2)	2.9 (1.2-5.9)	2.5 (1.1-5.2)	2.8 (1.2-5.6)	3.4 (1.3-7.2)	2.6 (1.1-5.4)	3.1 (1.2-6.2)	2.8 (1.2-5.6)
Median (IQR) age in years	43 (40-50)	41 (40-48)	40 (40-46)	43 (40-49)	44 (40-49)	43 (40-48)	42 (40-48)	43 (40-49)
Age (years)								
40–44	46,384 (58.4%)	25,639 (65.6%)	8,581 (70.9%)	39,466 (60.1%)	4,073 (55.8%)	4,662 (60.8%)	4,321 (61.2%)	8,814 (58.9%)
45–49	12,567 (15.8%)	5,732 (14.7%)	1,658 (13.7%)	11,340 (17.3%)	1,573 (21.6%)	1,444 (18.8%)	1,300 (18.4%)	2,818 (18.8%)
50–54	9,461 (11.9%)	3,901 (10%)	1,002 (8.3%)	7,429 (11.3%)	996 (13.6%)	862 (11.2%)	740 (10.5%)	1,712 (11.4%)
55–59	6,889 (8.7%)	2,454 (6.3%)	552 (4.6%)	4,585 (7%)	449 (6.2%)	432 (5.6%)	461 (6.5%)	980 (6.6%)
60–64	4,108 (5.2%)	1,333 (3.4%)	302 (2.5%)	2,879 (4.4%)	207 (2.8%)	263 (3.4%)	238 (3.4%)	632 (4.2%)
Sex								
Male	40,583 (51.1%)	21,494 (55%)	7,213 (59.6%)	32,249 (49.1%)	3,658 (50.1%)	4,186 (54.6%)	3,397 (48.1%)	7,492 (50.1%)
Female	38,823 (48.9%)	17,565 (45%)	4,882 (40.4%)	33,449 (50.9%)	3,640 (49.9%)	3,477 (45.4%)	3,663 (51.9%)	7,464 (49.9%)
Townsend quintile								
1 (least deprived)	12,766 (16.1%)	2,869 (7.3%)	661 (5.5%)	6,807 (10.4%)	675 (9.3%)	675 (8.8%)	1,117 (15.8%)	1,735 (11.6%)
2	10,118 (12.7%)	2,645 (6.8%)	851 (7%)	7,428 (11.3%)	759 (10.4%)	780 (10.2%)	1,138 (16.1%)	1,801 (12%)
3	15,212 (19.2%)	5,782 (14.8%)	1,466 (12.1%)	11,893 (18.1%)	996 (13.7%)	1,095 (14.3%)	1,323 (18.7%)	2,444 (16.3%)
4	21,441 (27%)	9,893 (25.3%)	2,199 (18.2%)	19,098 (29.1%)	1,581 (21.7%)	1,689 (22.1%)	1,471 (20.8%)	3,270 (21.9%)
5 (most deprived)	19,833 (25%)	17,851 (45.7%)	6,912 (57.2%)	20,434 (31.1%)	3,277 (45%)	3,417 (44.6%)	2,008 (28.5%)	5,701 (38.1%)
Region								
North East	669 (0.8%)	414 (1.1%)	255 (2.1%)	1,102 (1.7%)	28 (0.4%)	124 (1.6%)	143 (2%)	358 (2.4%)
North West	5,902 (7.4%)	7,016 (18%)	1,135 (9.4%)	4,756 (7.2%)	679 (9.3%)	904 (11.8%)	674 (9.6%)	1,424 (9.5%)
Yorkshire & Humber	1,024 (1.3%)	917 (2.4%)	147 (1.2%)	984 (1.5%)	137 (1.9%)	190 (2.5%)	195 (2.8%)	273 (1.8%)
East Midlands	1,843 (2.3%)	1,737 (4.5%)	124 (1%)	983 (1.5%)	196 (2.7%)	154 (2%)	110 (1.6%)	219 (1.5%)
West Midlands	19,200 (24.2%)	11,923 (30.7%)	1,934 (16%)	6,251 (9.5%)	1,176 (16.1%)	731 (9.5%)	840 (11.9%)	1,523 (10.2%)
East of England	4,734 (6%)	1,553 (4%)	1,026 (8.5%)	3,517 (5.4%)	330 (4.5%)	374 (4.9%)	373 (5.3%)	841 (5.6%)
South West	3,230 (4.1%)	1,415 (3.6%)	716 (5.9%)	3,722 (5.7%)	735 (10.1%)	595 (7.8%)	643 (9.1%)	1,297 (8.7%)
South Central	7,573 (9.5%)	2,894 (7.4%)	623 (5.2%)	7,119 (10.8%)	550 (7.5%)	850 (11.1%)	879 (12.5%)	1,323 (8.8%)
London	30,881 (38.9%)	9,591 (24.7%)	5,435 (45%)	32,055 (48.8%)	3,140 (43%)	3,224 (42.1%)	2,622 (37.2%)	6,777 (45.3%)
South East	4,296 (5.4%)	1,432 (3.7%)	678 (5.6%)	5,160 (7.9%)	326 (4.5%)	515 (6.7%)	576 (8.2%)	916 (6.1%)
Median (IQR) consultations in prior 12 months	3 (1-7)	4 (1-9)	5 (2-9)	3 (1-7)	4 (1-8)	3 (1-7)	3 (1-7)	3 (1-7)
BMI category *								
Underweight	1,615 (2.3%)	574 (1.7%)	267 (2.5%)	1,532 (2.7%)	83 (1.3%)	84 (1.3%)	144 (2.4%)	272 (2.1%)
Normal weight	30,778 (44.1%)	11,491 (33.9%)	4,782 (44.4%)	27,260 (47.3%)	2,395 (38.3%)	2,169 (33.5%)	3,056 (50.4%)	5,812 (45.4%)
Overweight	26,964 (38.6%)	14,117 (41.6%)	4,363 (40.5%)	21,183 (36.8%)	2,353 (37.6%)	2,587 (40%)	2,069 (34.1%)	4,593 (35.8%)
Obese	10,488 (15%)	7,727 (22.8%)	1,351 (12.6%)	7,626 (13.2%)	1,426 (22.8%)	1,627 (25.2%)	790 (13%)	2,136 (16.7%)
Smoking status *								
Non-smoker	49,203 (62.8%)	20,731 (53.8%)	5,413 (45.3%)	37,608 (58.1%)	2,554 (35.2%)	4,026 (53.3%)	3,352 (48%)	6,797 (46.1%)
Current smoker	10,267 (13.1%)	8,247 (21.4%)	3,345 (28%)	10,920 (16.9%)	2,631 (36.3%)	1,602 (21.2%)	1,605 (23%)	3,663 (24.9%)
Ex-smoker	18,904 (24.1%)	9,581 (24.8%)	3,196 (26.7%)	16,174 (25%)	2,066 (28.5%)	1,929 (25.5%)	2,020 (29%)	4,269 (29%)
Alcohol consumption *								
Not a heavy drinker	69,777 (96.7%)	34,806 (97.8%)	10,787 (97.1%)	56,979 (97.1%)	6,153 (93.1%)	6,421 (95.6%)	5,961 (95.3%)	12,614 (95.4%)
Heavy drinker	2,371 (3.3%)	784 (2.2%)	317 (2.9%)	1,720 (2.9%)	457 (6.9%)	293 (4.4%)	297 (4.7%)	603 (4.6%)

*Closest measure before baseline. BMI; body mass index, CPRD; Clinical Practice Research Datalink, IQR; interquartile range

We identified 102,316 influenza/ILI episodes recorded among 94,623 patients, and 560,860 ARI episodes among 421,349 patients. The rate of influenza/ILI was highest in the South Asian group (9.6 per 1,000 person-years) followed by the Black group (8.4 per 1,000 person-years) ( Table 4). In all ethnic groups the influenza/ILI rates were higher in women than men and decreased with age.

**Table 4.** Incidence rate ratios for influenza / influenza-like illness and acute respiratory infections.

			Influenza / influenza-like illness					Acute respiratory infections				
	Denom	PY per 1,000	Events	Rate per 1,000 PY	Crude IRR (95% CI)	Model 1 * IRR (95% CI)	Model 2 ** IRR (95% CI)	Events	Rate per 1,000 PY	Crude IRR (95% CI)	Model 1 * IRR (95% CI)	Model 2 ** IRR (95% CI)
Ethnicity by 5 categories												
White	3,271,115	15,249	87,486	5.7	1	1	1	509,256	33.4	1	1	1
South Asian	196,262	743	7,128	9.6	1.66 (1.62-1.71)	1.70 (1.66-1.75)	1.66 (1.62-1.71)	25,381	34.2	1.00 (0.98-1.01)	1.07 (1.05-1.09)	1.07 (1.05-1.09)
Black	157,075	629	5,308	8.4	1.46 (1.42-1.50)	1.48 (1.44-1.53)	1.37 (1.33-1.42)	16,489	26.2	0.76 (0.75-0.78)	0.81 (0.80-0.83)	0.83 (0.81-0.84)
Mixed	41,416	253	1,294	6.9	1.20 (1.13-1.28)	1.22 (1.15-1.30)	1.18 (1.11-1.26)	5,438	26.9	0.79 (0.76-0.82)	0.84 (0.81-0.88)	0.85 (0.82-0.88)
Other	69,440	160	1,110	5.1	0.89 (0.84-0.94)	0.90 (0.85-0.95)	0.88 (0.83-0.93)	4,296	21.5	0.62 (0.60-0.64)	0.65 (0.63-0.67)	0.67 (0.65-0.69)
Ethnicity by 16 categories												
British	2,315,904	11,206	64,329	5.7	1	1	1	381,294	34.0	1	1	1
Irish	27,339	118	710	6.0	1.04 (0.96-1.13)	1.06 (0.98-1.15)	1.04 (0.96-1.12)	4,097	34.8	1.02 (0.98-1.06)	1.03 (0.99-1.07)	1.05 (1.01-1.09)
Other White	232,692	770	4,424	5.7	0.99 (0.96-1.03)	1.03 (1.00-1.06)	0.99 (0.96-1.03)	18.531	24.1	0.68 (0.67-0.69)	0.72 (0.71-0.74)	0.75 (0.73-0.76)
Indian	79,409	32	210	6.6	1.71 (1.64-1.77)	1.71 (1.65-1.78)	1.70 (1.63-1.77)	970	30.5	0.92 (0.90-0.94)	0.96 (0.94-0.99)	0.98 (0.96-1.01)
Pakistani	39,059	28	210	7.6	1.94 (1.84-2.05)	1.99 (1.88-2.10)	1.90 (1.80-2.01)	646	23.5	1.30 (1.26-1.34)	1.42 (1.37-1.46)	1.28 (1.24-1.32)
Bangladeshi	12,095	27	189	6.9	2.16 (1.96-2.38)	2.26 (2.05-2.49)	2.09 (1.90-2.30)	761	27.9	1.25 (1.18-1.32)	1.41 (1.33-1.50)	1.35 (1.27-1.43)
Other Asian	65,699	55	362	6.6	1.35 (1.29-1.42)	1.37 (1.31-1.44)	1.33 (1.27-1.40)	1,387	25.2	0.81 (0.79-0.84)	0.86 (0.84-0.89)	0.89 (0.87-0.92)
Caribbean	44,722	317	3,123	9.8	1.29 (1.22-1.37)	1.27 (1.20-1.34)	1.17 (1.10-1.23)	10,209	32.2	0.77 (0.75-0.80)	0.79 (0.77-0.82)	0.78 (0.76-0.81)
African	84,355	145	1,617	11.2	1.57 (1.51-1.64)	1.60 (1.54-1.67)	1.47 (1.41-1.54)	6,519	45.0	0.69 (0.67-0.71)	0.75 (0.73-0.77)	0.75 (0.73-0.77)
Other Black	27,998	41	514	12.4	1.44 (1.35-1.55)	1.45 (1.35-1.56)	1.33 (1.24-1.43)	1,815	43.8	0.84 (0.81-0.88)	0.90 (0.86-0.94)	0.88 (0.84-0.92)
White + Black Caribbean	7,298	240	1,874	7.8	1.14 (0.99-1.32)	1.15 (1.00-1.33)	1.08 (0.94-1.25)	6,838	28.5	0.88 (0.82-0.95)	0.93 (0.86-1.01)	0.91 (0.84-0.98)
White + Black African	7,663	209	1,556	7.4	1.32 (1.14-1.52)	1.37 (1.18-1.58)	1.29 (1.12-1.49)	5,605	26.8	0.67 (0.61-0.73)	0.73 (0.67-0.80)	0.71 (0.65-0.78)
White + Asian	7,060	306	2,801	9.1	1.20 (1.03-1.39)	1.23 (1.06-1.43)	1.21 (1.04-1.41)	7,517	24.5	0.80 (0.74-0.87)	0.86 (0.79-0.94)	0.88 (0.81-0.96)
Other Mixed	14,956	113	951	8.4	1.15 (1.03-1.28)	1.18 (1.06-1.32)	1.13 (1.01-1.26)	3,367	29.7	0.73 (0.68-0.77)	0.77 (0.73-0.82)	0.78 (0.73-0.83)
Chinese	23,419	91	322	3.5	0.61 (0.54-0.68)	0.61 (0.54-0.68)	0.59 (0.53-0.66)	1,521	16.6	0.47 (0.44-0.50)	0.48 (0.45-0.51)	0.48 (0.46-0.51)
Other	46,021	161	972	6.0	1.04 (0.98-1.12)	1.05 (0.98-1.12)	1.01 (0.95-1.08)	3,917	24.3	0.69 (0.66-0.72)	0.73 (0.70-0.76)	0.74 (0.71-0.77)

*Model 1 is adjusted for 5-year age band, sex and year. All LRT p-values <0.001 **Model 2 is adjusted for 5-year age band, sex, year, Townsend deprivation quintile and region of residence. All LRT p-values <0.001 CI; confidence interval, IRR; incidence rate ratio, LRT; likelihood ratio test, PY; person-years

**Figure 2.**
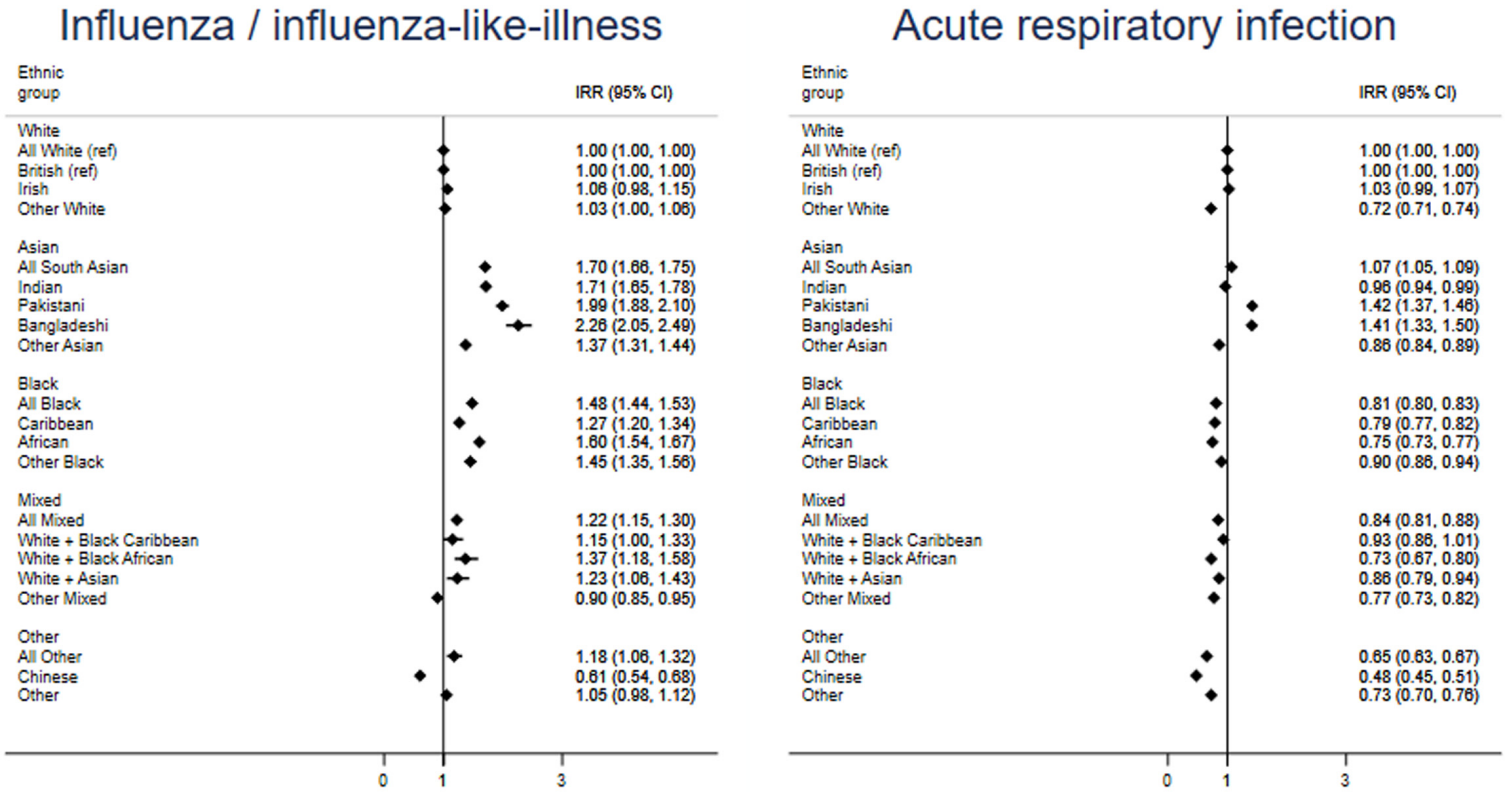
Ethnic differences in the incidence ratio risks of influenza / influenza-like illness and acute respiratory infections. The top row under each category shows the result for the 5 category breakdown of ethnicity with the rows listed beneath corresponding to the relevant 16 category breakdown of ethnicity. All White is the reference category for comparison of ethnicity in 5 categories. British is the reference category for comparison of ethnicity in 16 categories. Models were adjusted for 5-year age band, sex, and year.

After adjustment for age, sex and year, the incidence rate ratio (IRR) for influenza/ILI was higher for South Asian (1.70, 95% CI 1.66-1.75), Black (1.48, 95% CI 1.44-1.53), and Mixed (1.22, 95% CI 1.15-1.30) groups compared to the White group ( Figure 2, Table 4). When broken down into the 16 categories, the IRR for influenza/ILI was higher in all groups included in the South Asian, Black and Mixed broad ethnic classifications, with the highest IRR in the Bangladeshi group (2.26, 95% CI 2.05-2.49). After additional adjustment for deprivation and region, results remained similar.

For ARI, the IRR was higher in the South Asian group (1.07, 95% CI 1.05-1.09) when compared to the White group, but lower in the Black (0.81, 95% CI 0.80-0.83), Mixed (0.84, 95% CI 0.81-0.88) and Other (0.65, 95% CI 0.63-0.67) groups. Using the 16 categories, the IRR for ARI was only higher for the Pakistani (1.42, 95% CI 1.37-1.46) and Bangladeshi (1.41, 95% CI 1.33-1.50) groups when compared with the White British group.

## Discussion

We showed an increased rate of influenza/ILI among Black, South Asian and Mixed groups based on clinical diagnoses following healthcare attendance. Specifically, those of Indian, Pakistani, Bangladeshi and African ethnicity had the highest rate compared to the White British group. When using our broader outcome of ARI, we only found an increased rate in the South Asian group with decreased rates in Black, Mixed and Other groups.

Our results suggest the risk of clinical influenza/ILI diagnosis risk differs between White and non-White groups. Such findings are consistent with studies of other acute viral respiratory infections including those which investigated the ethnic disparities in severe influenza outcomes, particularly during the 2009 H1N1 pandemic ^10, 11^ as well as studies of COVID-19 infection risk and severe outcomes ^3^.

Our study was conducted among patients not eligible for vaccination, and so disparities cannot be explained by differences in vaccine uptake or effectiveness: there are potentially even larger ethnic differences in influenza incidence among those eligible for influenza vaccine due to inequalities in chronic disease patterns. Since social mixing and household contact are important considerations for influenza/ILI transmission our findings are relevant to the whole population. People of non-white ethnicity tend to live in larger, multi-generational households with extended kinship and social networks ^14, 15^. Therefore, understanding ethnic disparities in respiratory infections across both high- and low-risk populations remains important for preventing hospitalizations.

Here we have presented results of a large population-based cohort study using nationally representative data. Excluding patients eligible for influenza vaccination due to chronic medical conditions should have reduced confounding. Nevertheless, our study may be impacted by some limitations. Under-diagnosis of health conditions may differ by ethnicity, with people from some ethnic groups less likely to be excluded from our study population but more likely to have an undiagnosed, and therefore unmanaged condition, which may affect influenza risk. Ethnicity may be less well recorded in GP records for individuals without a chronic condition requiring frequent consultation, but financial incentivization between 2006–2011 boosted completion in GP records. Using hospital data boosted the completeness of ethnicity recording in our study population from 74% to 88%.

Influenza/ILI identification in our study was based on clinical diagnosis following healthcare attendance. Clinically identified influenza/ILI depends not only on attendance but also clinical coding practices, both of which may be associated with ethnicity. However, our results are consistent with other studies which used laboratory-confirmed measures of acute viral respiratory infections ^3, 10, 11^. Our differing results for influenza/ILI and ARI outcomes may be attributable to the lack of specificity of ARI codes for influenza. We excluded individuals with known risk factors for influenza; it may be that other conditions are relevant risk factors for ARI generally.

Ethnic inequalities in the incidence of respiratory infections could arise because of differences in risk of exposure. Differences in exposure risk may be driven by factors such as occupation, including working in frontline high-exposure occupations (including healthcare settings), and household composition, with large multigenerational households more common in non-White ethnic groups ^16^, as well as inequalities in access to care. Results from analysis of ethnic inequalities in access to care are mixed with the reasons for any inequalities complex, and likely due to multiple interlinked factors including; different cultural approaches to health, experiences of discrimination, and language barriers ^17, 18^. Potentially ethnic differences in influenza/ILI incidence could be greater than we have shown depending on the extent of access to care inequality. Unequal access to treatments will also affect the likelihood of adverse outcomes after infection.

We excluded children who are a key driver for influenza transmission; examining ethnic inequalities for infection risk in children is an area for future research.

The COVID-19 pandemic has drawn attention to the ethnic inequalities in infection risk. Ethnic disparities in outcomes have been previously highlighted, during the 2009 H1N1 influenza pandemic as well as for seasonal influenza ^10, 11^. Our study found that ethnic inequalities are also present for seasonal influenza/ILI. This reinforces the urgency of addressing lower influenza, and now COVID-19, vaccine uptake among minority ethnic groups ^19^. We suggest targeted public health interventions are implemented to facilitate increased vaccine uptake in non-White ethnic groups.

## Data availability 

### Source data

The patient data used in this study are supplied from Clinical Practice Research Datalink (CPRD; www.cprd.com) but restrictions apply to the availability of these data, which were obtained under licence from the UK Medicines and Healthcare products Regulatory Agency, and so are not publicly available. For re-using these data, an application must be made directly to CPRD. Instructions for how to submit an application and the conditions under which access will be granted are explained at https://www.cprd.com/research-applications.

### Ethical approval

The study was approved by the CPRD Independent Scientific Advisory Committee (Protocol number: 19_209A2) and the London School of Hygiene and Tropical Medicine Research Ethics Committee (Reference: 17894).
