# Supplementary material for: Ethnic differences in the incidence of clinically diagnosed influenza: an England population-based cohort study 2008-2018
Source: Wellcome Open Res. 2021 Jun 10;6:49. Originally published 2021 Mar 4. [Version 3] doi: 10.12688/wellcomeopenres.16620.3 (PMC8136253; doi:10.12688/wellcomeopenres.16620.3)
Supplement: Peer review history

# Peer review: Reviewer response for version 3

**DOI:** 10.21956/wellcomeopenres.18707.r44272

**Published:** 2021-06-10

**Authors:** Socorro Lupisan

**Affiliations:** 1 Department of Health, Research Institute for Tropical Medicine, Muntinlupa, Philippines

Thank you for the response and modifications done. I have no more comments.

Is the work clearly and accurately presented and does it cite the current literature?

Yes

If applicable, is the statistical analysis and its interpretation appropriate?

Partly

Are all the source data underlying the results available to ensure full reproducibility?

Yes

Is the study design appropriate and is the work technically sound?

Yes

Are the conclusions drawn adequately supported by the results?

Yes

Are sufficient details of methods and analysis provided to allow replication by others?

Partly

Reviewer Expertise:

Clinical Epidemiology/Family Medicine Specialist

I confirm that I have read this submission and believe that I have an appropriate level of expertise to confirm that it is of an acceptable scientific standard.

# Peer review: Reviewer response for version 3

**DOI:** 10.21956/wellcomeopenres.18707.r44368

**Published:** 2021-06-10

**Authors:** Benjamin J. Cowling

**Affiliations:** 1 WHO Collaborating Centre for Infectious Disease Epidemiology and Control, School of Public Health, The University of Hong Kong, Hong Kong, China

No further comments

Is the work clearly and accurately presented and does it cite the current literature?

Yes

If applicable, is the statistical analysis and its interpretation appropriate?

Yes

Are all the source data underlying the results available to ensure full reproducibility?

Partly

Is the study design appropriate and is the work technically sound?

Yes

Are the conclusions drawn adequately supported by the results?

Yes

Are sufficient details of methods and analysis provided to allow replication by others?

Yes

Reviewer Expertise:

NA

I confirm that I have read this submission and believe that I have an appropriate level of expertise to confirm that it is of an acceptable scientific standard.

# Peer review: Reviewer response for version 2

**DOI:** 10.21956/wellcomeopenres.18537.r43676

**Published:** 2021-05-19

**Authors:** Socorro Lupisan

**Affiliations:** 1 Department of Health, Research Institute for Tropical Medicine, Muntinlupa, Philippines

1. The article is much better written now with the objective of determining incidence of influenza in different racial groups.
2. In the results section, who are the "Other Asian"? This is relevant to me as an Asian. Where will Filipinos, or other Asians fall under, other than Chinese which is specified? Does it mean, if not South Asian, they are "Other Asian"?
3. Region of residence will not be relevant to readers who are not familiar, unless this is classified as urban, peri-urban or rural, which can be relatable. 
4. Still, the tables are too long. No comparison for significant difference has been done. May be added as supplement.

Is the work clearly and accurately presented and does it cite the current literature?

Yes

If applicable, is the statistical analysis and its interpretation appropriate?

Partly

Are all the source data underlying the results available to ensure full reproducibility?

Yes

Is the study design appropriate and is the work technically sound?

Yes

Are the conclusions drawn adequately supported by the results?

Yes

Are sufficient details of methods and analysis provided to allow replication by others?

Partly

Reviewer Expertise:

Clinical Epidemiology/Family Medicine Specialist

I confirm that I have read this submission and believe that I have an appropriate level of expertise to confirm that it is of an acceptable scientific standard.

# Response

**Published:** 2021-06-04

**Authors:** Jennifer Davidson

**Affiliations:** London School of Hygiene & Tropical Medicine, UK

Dear Dr Lupisan,

 Thank you for re-reviewing our article and for your positive feedback. 

 In response to your specific points:

1. In the results section, who are the "Other Asian"? This is relevant to me as an Asian. Where will Filipinos, or other Asians fall under, other than Chinese which is specified? Does it mean, if not South Asian, they are "Other Asian"?
*Response: To clarify, we based our categories of ethnicity on those used in the UK Census 2001, which was the most recent census prior to our study period. The 2001 Census groupings included an overall Asian category with sub-groupings of ‘Indian’, ‘Pakistani’, ‘Bangladeshi’ and 'Other Asian', and then collected 'Chinese' or 'Other' separately https://history.blog.gov.uk/2019/03/07/50-years-of-collecting-ethnicity-data/. In order to make our results clinically interpretable, we grouped all South Asian ethnicities together (as 'Indian', 'Pakistani', 'Bangladeshi' and 'Other South Asian'). Those reporting other specified Asian ethnicities that were not South Asian would form part of the 'Other' group, i.e. someone who was documented as Filipino would be grouped under 'Other'. We have updated the labels in our graphs and tables from 'Other Asian' to 'Other South Asian' for clarity.  *
2. Region of residence will not be relevant to readers who are not familiar, unless this is classified as urban, peri-urban or rural, which can be relatable.
*Response: We used the 10 regional breakdowns in England available in CPRD, which are categorised geographically. There is substantial variation within each region so they are not able to be classified as urban, peri-urban and rural. However, our inclusion of the Townsend quintile gives further information about area-level deprivation, which is more relevant to our study question.*
3. Still, the tables are too long. No comparison for significant difference has been done. May be added as supplement.
*Response: We agree that the tables are long, but we think the* *importance of the information shown outweighs the issue of length.* *Wellcome Open Research do not permit supplementary material so we are unable to add the tables as an appendix. We acknowledge that we have not added a comparison of significant differences to the tables; due to the size of our dataset p-values can be uninformative.*

# Peer review: Reviewer response for version 1

**DOI:** 10.21956/wellcomeopenres.18323.r42944

**Published:** 2021-03-22

**Authors:** Socorro Lupisan

**Affiliations:** 1 Department of Health, Research Institute for Tropical Medicine, Muntinlupa, Philippines

The database is robust and the study is well designed. 

 But the authors need to address the following issues re methods and analysis:

1. The ethnic grouping is the major variable in this study. It is not clear cut. For South Asians, what other Asian nationalities are included? For Other, are these Chinese and other Asians? Please specify.
2. Describe the health care delivery system in the UK, so readers will understand your sample population that had access to health services. Anyone can avail of free medical services? Is there unequal access? Is access to health care a risk variable?
3. When the groupings are clear, it is best to compare these major groups as to socio demographic profile and establish any significant difference (or no difference) among the ethnic groups. (Show the p-values): age group 40-49,50-59, 60+, Townsend quintile 4,5 (yes/no), region is unclear to me- best to say urban, periurban or rural.
4. More than just establishing the incidence of influenza, ILI and ARI by ethnicity, you also can establish the risk factors for such by doing #3.
5. Similar observations have been published for 2009 H1N1 and COVID-19. The conclusion should have a stronger recommendation for health care for ethnic groups at risk.
6. There are too many tables and figures. Important ones are Figure 1, simplified Table 1 showing statistical significant differences, Figure 3 with the revised or clear groupings.

Is the work clearly and accurately presented and does it cite the current literature?

Yes

If applicable, is the statistical analysis and its interpretation appropriate?

Partly

Are all the source data underlying the results available to ensure full reproducibility?

Yes

Is the study design appropriate and is the work technically sound?

Yes

Are the conclusions drawn adequately supported by the results?

Yes

Are sufficient details of methods and analysis provided to allow replication by others?

Partly

Reviewer Expertise:

Clinical Epidemiology/ Family Medicine Specialist

I confirm that I have read this submission and believe that I have an appropriate level of expertise to confirm that it is of an acceptable scientific standard, however I have significant reservations, as outlined above.

# Response

**Published:** 2021-04-08

**Authors:** Jennifer Davidson

**Affiliations:** London School of Hygiene & Tropical Medicine, UK

Dear Dr Lupisan, 

 Thank you for your detailed response and helpful comments on our manuscript. Below are our responses to each of your questions/points:  

***Point 1: The ethnic grouping is the major variable in this study. It is not clear cut. For South Asians, what other Asian nationalities are included? For Other, are these Chinese and other Asians? Please specify.***

 Response to point 1: We have listed the five categories used in the variables section of the methods with the relevant 16 category grouping in brackets after each of the five categories. We have now added text to state this is how the groups correspond to each other.  

***Point 2: Describe the health care delivery system in the UK, so readers will understand your sample population that had access to health services. Anyone can avail of free medical services? Is there unequal access? Is access to health care a risk variable?***

 Response to point 2: We have added further detail on how data populate CPRD and HES as well as the setup of the NHS in the UK to the data sources section of the methods to aid international reader understanding “CPRD GOLD and Aurum collect records from >35 million patients registered at with National Health Service (NHS) general practitioners. HES APC data are collated from inpatient care at all NHS hospitals in England. The NHS is a universal health system publicly funded through general taxation which is accessible to all UK residents, although there is an annual surcharge for people who move to the UK”.   

 We have also expanded our discussion of ethnic differences in healthcare attendance in our limitations to include more detail the issues of inequalities in access to care. However, there is no way for us to directly measure access within the dataset other than consultation frequency, which we have included in our baseline characteristics table.  

***Point 3: When the groupings are clear, it is best to compare these major groups as to socio demographic profile and establish any significant difference (or no difference) among the ethnic groups. (Show the p-values): age group 40-49,50-59, 60+, Townsend quintile 4,5 (yes/no), region is unclear to me- best to say urban, periurban or rural.***

 Response to point 3: We agree that if conducting an analysis of overall risk factors for a clinical diagnosis of influenza/ILI by ethnicity it would be of interest to present statistical differences for baseline characteristics by ethnic group. However, we only intended to describe the baseline characteristics of the study population as we are not investigating the risk factors for ethnic differences in ILI. Additionally, with large electronic health record datasets p-values can be uninformative. We have expanded the description of our cohort in the text at the start of our results section.  

 We used anonymised data with location of residence in the dataset available as regions of England, which do not correspond to rural/urban status. The regions of England are well recognised within the country and are the geography with public health is organised. Flu circulation may vary regionally with the ethnic profile of the population also varying regionally, so we considered region as a confounder.  

***Point 4: More than just establishing the incidence of influenza, ILI and ARI by ethnicity, you also can establish the risk factors for such by doing #3.***

 Response to point 4: Thank you for this suggestion. The aim of our analysis was to investigate whether the incidence of influenza/ILI varied by ethnic group, accounting for key confounding factors. This was a particularly relevant question for public health professionals to answer following the COVID-19 pandemic. We did not aim to investigate the individual risk factors associated with the ethnic variation in influenza/ILI incidence, for which a different analysis would be appropriate.   

***Point 5: Similar observations have been published for 2009 H1N1 and COVID-19. The conclusion should have a stronger recommendation for health care for ethnic groups at risk.***

 Response to point 5: We have strengthened our concluding remarks to “The COVID-19 pandemic has drawn attention to the ethnic inequalities in infection risk. Ethnic disparities in outcomes have been previously highlighted, during the 2009 H1N1 influenza pandemic as well as for seasonal influenza. Our study found that ethnic inequalities are also present for the incidence of clinically diagnosed seasonal influenza/ILI. This reinforces the urgency of addressing lower influenza, and now COVID-19, vaccine uptake among minority ethnic groups. We suggest targeted public health interventions are implemented to facilitate increased vaccine uptake in non-White ethnic groups.” 

***Point 6: There are too many tables and figures. Important ones are Figure 1, simplified Table 1 showing statistical significant differences, Figure 3 with the revised or clear groupings.***

 Response to point 6: We used two breakdowns for ethnicity – one with 5 groupings and one with 16 groupings, while this unfortunately results in long tables we think it is important to show the baseline characteristics, incidence rates and IRRs for all ethnic groups and both outcomes used (influenza/ILI and ARI). Inclusion of these data aids reader understanding of our key findings and interpretation of the results. Ideally, we would use a supplementary appendix to display the large tables, however, Wellcome Open Research’s format requires all tables and figures be in the main text with supplementary material not permitted. We have updated Figure 3 (now named Figure 2) footnote to explain the groupings presented and removed Figure 2.

# Peer review: Reviewer response for version 1

**DOI:** 10.21956/wellcomeopenres.18323.r42945

**Published:** 2021-03-08

**Authors:** Benjamin J. Cowling

**Affiliations:** 1 WHO Collaborating Centre for Infectious Disease Epidemiology and Control, School of Public Health, The University of Hong Kong, Hong Kong, China

This is a nice study using electronic health records, with clear methodology and findings.

 Influenza/ILI in CPRD is mainly ILI, so I am not sure your conclusion "Our results suggest influenza infection risk..." might be fairer to discuss ILI than influenza virus infection?

 To what extent could access to care also affect comparisons? You are not studying incidence of infections directly, but medically-attended illnesses? On a related note, if sick-notes are required in service sector but less frequently for white collar professions, would it lead to greater consultations among lower income professions?

 I saw 12 references in the bibliography and wondered if there might be more (additional) similar studies that it would be relevant to cite here?

Is the work clearly and accurately presented and does it cite the current literature?

Yes

If applicable, is the statistical analysis and its interpretation appropriate?

Yes

Are all the source data underlying the results available to ensure full reproducibility?

Partly

Is the study design appropriate and is the work technically sound?

Yes

Are the conclusions drawn adequately supported by the results?

Yes

Are sufficient details of methods and analysis provided to allow replication by others?

Yes

Reviewer Expertise:

Infectious disease epidemiology

I confirm that I have read this submission and believe that I have an appropriate level of expertise to confirm that it is of an acceptable scientific standard.

# Response

**Published:** 2021-04-08

**Authors:** Jennifer Davidson

**Affiliations:** London School of Hygiene & Tropical Medicine, UK

Dear Professor Cowling, 

 Thank you for your positive feedback and helpful comments. Below are our responses to each of your questions/points:  

***Point 1: Influenza/ILI in CPRD is mainly ILI, so I am not sure your conclusion "Our results suggest influenza infection risk..." might be fairer to discuss ILI than influenza virus infection?***

 Response to point 1: Thank you for highlighting this, it would indeed be fairer to discuss ILI. We have updated the relevant text to “Our results suggest the risk of clinical influenza/ILI diagnosis differs between White and non-White groups”. We have used “influenza/ILI” in other sections of our discussion, so think the continued use of this phase adds consistency and acknowledges that some infections will be due to the influenza virus while others are not.  

***Point 2: To what extent could access to care also affect comparisons?***

 Response to point 2: We have expanded our discussion of ethnic inequalities in access to care in our limitations section to include further consideration on inequalities in access to care: “Ethnic inequalities in the incidence of respiratory infections could arise because of differences in risk of exposure. Differences in exposure risk may be, driven by factors such as occupation, including working in frontline high-exposure occupations (including healthcare settings), and household composition, with large multigenerational households more common in non-White ethnic groups(16), as well as inequalities in access to care. Results from analysis of ethnic inequalities in access to care are mixed with the reasons for any inequalities complex, and likely due to multiple interlinked factors including; different cultural approaches to health, experiences of discrimination, and language barriers(17,18). Potentially ethnic differences in influenza/ILI incidence could be greater than we have shown depending on the extent of access to care inequality. Unequal access to treatments will also affect the likelihood of adverse outcomes after infection.” 

***Point 3: You are not studying incidence of infections directly, but medically-attended illnesses?***

 Response to point 3: We have rephased the text in several places to emphasise that our incidence findings are based on illnesses which were medically-attended, including in the introduction where we state our study aim, in the opening sentence of our discussion, and in our limitations section of the discussion.  

***Point 4: On a related note, if sick-notes are required in service sector but less frequently for white collar professions, would it lead to greater consultations among lower income professions?***

 Response to point 4: There are likely differences in the requirement of sick-notes within different professions in England. We are not able to comment on this directly but we did adjust for socio-economic status in our analyses to minimise the impact of any such differences.  

***Point 5: I saw 12 references in the bibliography and wondered if there might be more (additional) similar studies that it would be relevant to cite here?***

 Response to point 5: We have expanded our bibliography to 19 references to include additional relevant literature. 
